# Complement and Non-Complement Binding Anti-HLA Antibodies Are Differentially Detected with Different Antigen Bead Assays in Renal Transplant Recipients

**DOI:** 10.3390/jcm12247733

**Published:** 2023-12-17

**Authors:** Konstantinos Ouranos, Manolis Panteli, Georgios Petasis, Marianthi Papachristou, Artemis Maria Iosifidou, Myrto Aikaterini Iosifidou, Aikaterini Anastasiou, Margarita Samali, Maria Stangou, Ioannis Theodorou, Georgios Lioulios, Asimina Fylaktou

**Affiliations:** 1 Department of Medicine, Houston Methodist Research Institute, Houston, TX 77030, USA; kostassky@hotmail.com; 2 School of Medicine, Aristotle University of Thessaloniki, 45636 Thessaloniki, Greece; pantelimanolis@gmail.com (M.P.); marpapach@hotmail.com (M.P.); ios.artemis.2000@gmail.com (A.M.I.); ios.myrtv.2002@gmail.com (M.A.I.); pter43@yahoo.gr (G.L.); 3 National Peripheral Histocompatibility Center, Department of Immunology, Hippokration Hospital, 54642 Thessaloniki, Greece; katanasta3@gmail.com (A.A.); fylaktoumina@gmail.com (A.F.); 4 1st Department of Nephrology, Hippokration Hospital, 54642 Thessaloniki, Greece; 5 Laboratoire d’Immunologie, Hôpital Robert Debré, 75019 Paris, France; ioannis.theodorou@aphp.fr

**Keywords:** anti-HLA antibodies, transplant, complement binding, Immucor, One-Lambda, Luminex, donor-specific antibodies

## Abstract

Two semi-quantitative, Luminex-based, single-antigen bead (SAB) assays are available to detect anti-HLA antibodies and evaluate their reactivity with complement binding. Sera from 97 patients with positive panel reactive antibody tests (>5%) were analyzed with two SAB tests, Immucor (IC) and One-Lambda (OL), for anti-HLA antibody detection and the evaluation of their complement-binding capacity. IC detected 1608/8148 (mean fluorescent intensity (MFI) 4195 (1995–11,272)) and 1136/7275 (MFI 6706 (2647–13,184)) positive anti-HLA class I and II specificities, respectively. Accordingly, OL detected 1942/8148 (MFI 6185 (2855–12,099)) and 1247/7275 (MFI 9498 (3630–17,702)) positive anti-HLA class I and II specificities, respectively. For the IC assay, 428/1608 (MFI 13,900 (9540–17,999)) and 409/1136 (MFI 11,832 (7128–16,531)) positive class I and II specificities bound C3d, respectively. Similarly, OL detected 485/1942 (MFI 15,452 (9369–23,095)) and 298/1247 (MFI18,852 (14,415–24,707)) C1q-binding class I and II specificities. OL was more sensitive in detecting class I and II anti-HLA antibodies than IC was, although there was no significant difference in the number of class II specificities per case. MFI was higher for complement vs. non-complement-binding anti-HLA antibodies in both assays. Both methods were equal in detecting complement-binding anti-HLA class I antibodies, whereas the C3d assay was more sensitive in detecting complement-binding anti-HLA class II antibodies.

## 1. Introduction

Renal transplantation is considered to be the treatment of choice for end-stage renal disease (ESRD) [1,2]. It is now well established that antibody-mediated rejection (AMR) is frequent in patients with new-onset kidney allograft dysfunction [3,4]. The detrimental effects of AMR can be attributed to preformed or de novo donor-specific antibodies (dnDSAs) arising after transplantation [5]. Extensive studies have been conducted regarding the target and nature of these antibodies, and whether or not the proper identification of the culprit graft antigens could provide more information regarding treatment strategies and prognosis, since not all antibodies detected are pathogenic. 

Antibodies against human leucocyte antigen (HLA) molecules have been found to play a pivotal role in AMR and subsequent graft failure [6,7,8]. To date, two semi-quantitative, Luminex-based, single-antigen bead (SAB) assays are available to detect the presence and amount of anti-HLA antibodies by measuring the mean fluorescent intensity (MFI) of serum samples [9]. Several studies have reported a correlation between higher MFI values and worse prognostic outcomes [10,11].

Recent insights regarding the pathogenicity and mechanism of HLA-associated AMR have found that complement binding by anti-HLA antibodies is more predictive of adverse graft outcomes as it results in downstream cascade activation, with terminal complement elements mediating tissue damage and graft loss [12,13]. Loupy et al. was the first to assess a group of 1016 renal transplant patients and found that the presence of complement-binding anti-HLA antibodies was associated with a worse 5-year graft survival rate when compared to the presence of non-complement-binding anti-HLA antibodies, or to the absence of anti-HLA antibodies [14]. Two SAB assays are currently available for the detection of C1q- or C3d-binding anti-HLA antibodies.

An evaluation of the two SAB assays for anti-HLA antibody determination and further characterization of their ability to fix complement components could impact diagnostic practices in transplant recipients. We performed a study to investigate the differences in the detection of anti-HLA antibodies between Immucor (IC) and One-Lambda (OL), and to evaluate the complement binding capacity of the antibodies detected using each method.

## 2. Materials and Methods

### 2.1. Patients

Patients with serum samples for anti-HLA antibody examination in the National Peripheral Histocompatibility Center of Hippokration Hospital of Thessaloniki, from June 2019 to December 2021, were eligible for inclusion in the study. The patients were either kidney transplant recipients (routinely tested annually, or after clinical or laboratory evidence of rejection) or patients on dialysis in a waiting list for kidney transplant, tested quarterly per year. 

The protocol of the study was approved by the Ethics Review Board of Hippokration Hospital of Thessaloniki (No. of approval 291/21.02.2019). Informed consent was obtained from each patient before enrollment in the study. The study was conducted in accordance with the Declaration of Helsinki.

### 2.2. Detection of Anti-HLA Antibodies

In order to quantify anti-HLA class I and class II antibodies, sera from 97 patients with a positive panel reactive antibody test (>5%) were further analyzed with two SAB tests, LIFECODES^®^ Single Antigen Assay (Immucor GTI Diagnostics, Inc, Waukesha, WI, USA) and LABScreen Single Antigen Bead Assay (One-Lambda, Thermo Fisher, West Hills, CA, USA), in accordance with the manufacturer’s instructions, as described below. Moreover, the sera were examined for complement binding using assays provided by the same companies, LIFECODES^®^ C3d Detection Assay (Immucor GTI Diagnostics, Inc, Waukesha, WI, USA) and C1q-SAB assays (One Lambda, Thermo Fisher, West Hills, CA, USA). All sera were preserved at −80 °C until testing. Ethylenediaminetetraacetic acid (EDTA) pretreatment was performed to prevent the prozone effect. Analysis was performed on a Luminex 100 flow multicolor cytometer (LABScan^TM^100, Luminex, Austin, TX, USA), and normalized MFI values were recorded for each bead. The analysis was carried out with One Lambda: Fusion 4.2 and since 2020 Fusion 4.4 and Immucor software: Mach IT 1.3.1. MFI values greater than 1000 were considered positive for anti-HLA antibodies (normalized MFI values for OL and raw MFI values for IC). Complement binding assays (performed without EDTA pretreatment) were considered positive when MFI values were greater than 500. Only common beads between the two assays were included in the statistical analysis (Supplementary Table S1).

### 2.3. Statistical Analysis

Statistical analysis was performed with SPSS.25 for Windows (IBM, Armonk, NY, USA). Categorical variables were reported as relative and absolute frequencies. All data were reported as the median (25th–75th percentile) for continuous variables unless otherwise indicated. Differences between groups were evaluated using the Chi-square test for categorical variables. Comparisons of continuous variables between two groups were evaluated with the Mann–Whitney U test or Wilcoxon test for independent or related samples, respectively. A two-way analysis of variance (ANOVA) was used to evaluate the effect of multiple variables in comparisons among subgroups. Spearman’s correlation was used to associate MFI values between the two methods. A two-tailed *p* value < 0.05 was considered statistically significant. 

For PCA analysis, all reactions observed for all common antigen-coated beads from the two vendors were taken into account. We created lists with all available reactions even if they were considered negative according to the vendor’s instructions. We considered about 60 common beads from each patient, thus “erasing” the antigen specificity of the reagents, but “inflating” the number of reactions according to their complement binding behavior, compared to those under simple antigen detection. This helps better understand the overall complement binding detection properties of the reagents with a simple multidimensional descriptive method.

## 3. Results

### 3.1. Patient Demographics

Characteristics of the patients included in the study are summarized in Table 1. Briefly, out of the 97 patients included, 50 (52%) were male, and the mean age of the entire cohort was 29 years. The number of patients that received a transplant was 72 (74.2%), of whom 42 (43.2%), 26 (26.8%), and 4 (4.1%) underwent live, deceased, or live and deceased donor transplantation, respectively.

### 3.2. Comparison of the Two Non-Complement SAB Methods

#### Positive Screening Tests According to the Two Methods

In 60 cases, both SAB assays detected positive antibodies for at least one class I specificity; in another 19 cases, at least one positive specificity was detected with only one assay (11 only with IC and 8 only with OL), and the rest of the 18 cases were negative for anti-HLA class I antibodies with both assays. In the 79 cases with at least one positive specificity, the number of anti-HLA class I specificities per case was greater for the OL than for the IC assay, at 25 (9–34) vs. 18 (7–28), respectively (*p* < 0.001). For class II specificities, 60 cases were positive with both assays, 9 cases were positive with only one assay (5 only with IC and 4 only with OL) and 28 cases were negative for anti-HLA class II antibodies in both assays. In the 69 cases with at least one positive specificity, 16 (10–25) vs. 16 (8–23) specificities were detected as positive per case for OL and IC, respectively (*p =* 0.057).

### 3.3. MFI Value Differences between Positive Specificities

The SAB assay using IC detected 1608 positive anti-HLA class I specificities out of the 8148 tested, and 1136 positive anti-HLA class II specificities out of the 7275 tested. Median MFI values were 4195 (1995–11,272) for class I and 6706 (2647–13,184) for class II anti-HLA antibodies. Accordingly, the SAB assay using OL detected a greater number of positive specificities than did that using IC, with 1942 positive anti-HLA class I specificities out of the 8148 tested, and 1247 positive anti-HLA class II specificities out of the 7275 tested, and with a median MFI value of 6185 (2855–12,099) for class I and of 9498 (3630–17,702) for class II, respectively. Moreover, 5977 class I specificities were negative with both methods and comprised 91.4% of 6540 negative IC class I specificities and 92.7% of 6206 negative OL class I specificities (chi square = 4231, *p* <0.001), while 5693 class II specificities were negative with both methods and comprised 92.7% of 6139 negative IC class II specificities and 94.4% of 6028 negative OL class II specificities (chi square = 2699, *p* < 0.001).

**Figure 1:**
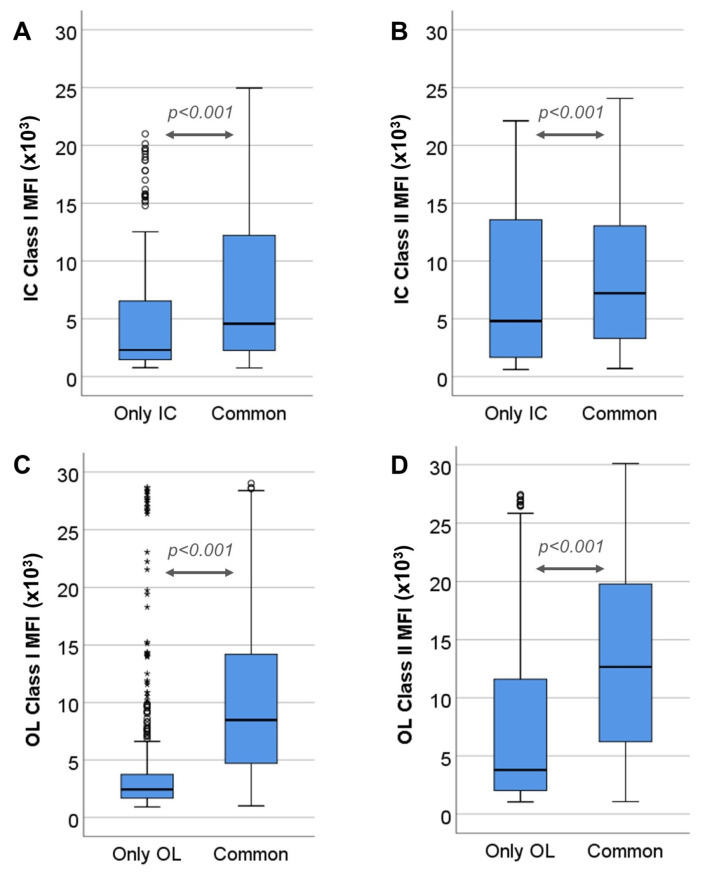
Comparison of MFI values between specificities detected using only one of the assays and those detected using both (**A**). MFI values, yielded via Immucor (IC), of class I specificities positive compared to specificities detected as positive using both methods (Common) (**B**). MFI values, detected using IC, of class II specificities detected as positive only via IC compared to specificities detected as positive using both methods (Common) (**C**). MFI values, detected using One-Lambda (OL), of class I specificities determined as positive only using OL compared to specificities determined as positive using both methods (Common) (**D**). MFI values, detected using OL, of class II specificities detected as positive only in OL compared to specificities positive detected as positive using both methods (Common). Circles in the diagram denote outliers laying 1.5–3 times away from the upper limit of the corresponding box. Asterisks denote outliers laying more than 3 times away from the upper limit of the corresponding box.

Out of the 1608 positive IC class I specificities, 1379 (85.7%) were also positive with OL, while 229 (14.2%) were detected only via IC (chi square = 4231, *p* < 0.001), with a median MFI value of 4577 (2254–12,242) vs. 2298 (1463–6537), respectively (*p* < 0.001) (Figure 1A). Regarding IC class II specificities, 801 out of 1136 (70.5%) were positive with both methods and 335 (29.5%) were positive only with IC (chi square = 2699, *p* <0.001), with a median MFI value of 7210 (3298–13,068) vs. 4804 (1656–13,658), respectively (*p* < 0.001) (Figure 1B). 

In the same way, the percentage of common positive class I specificities as detected via OL was 71% (1379 out of 1942), while 563 (28.9%) were detected only via OL, with median MFI value 8474 (4706–14,149) vs. 2437 (1676–3749), respectively (*p* < 0.001) (Figure 1C). The percentage of common positive OL class II specificities was 64.2% (801 out of 1247) while 446 (35.7%) were positive only with OL, with a median MFI value of 12,652 (6222–19,845) vs. 3787 (2117–11,603), respectively (*p* < 0.001) (Figure 1D).

**Figure 2:**
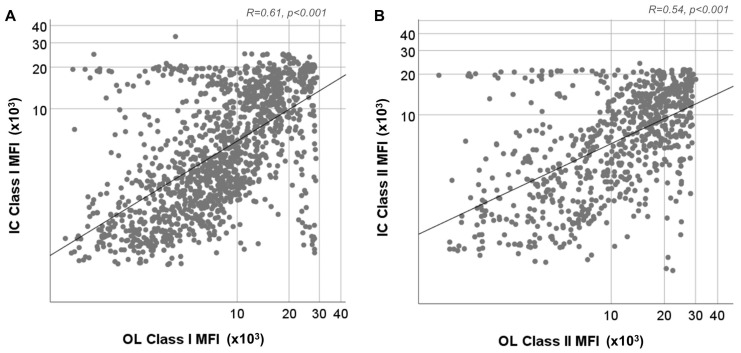
Correlation of MFI values of specificities detected as positive using both assays. (**A**) Spearman’s correlation of MFI values of class I specificities determined via Immucor (IC) with MFI values of class I specificities determined using One-Lambda (OL). (**B**) Spearman’s correlation of MFI values of class II specificities determined via IC with MFI values of class II specificities determined via OL.

The linear correlation between positive specificities for both methods is shown in Figure 2. Class I and II anti-HLA antibody MFI values were significantly positively correlated (r = 0.61, *p* < 0.001 and r = 0.54, *p* = 0.001 for class I and II anti-HLA antibodies, respectively). However, not all specificities had significant correlations in their MFI values between the two assays, as shown in Supplementary Table S2.

### 3.4. MFI Value Comparisons of Complement- and Non-Complement-Detecting SAB Methods

**Figure 3:**
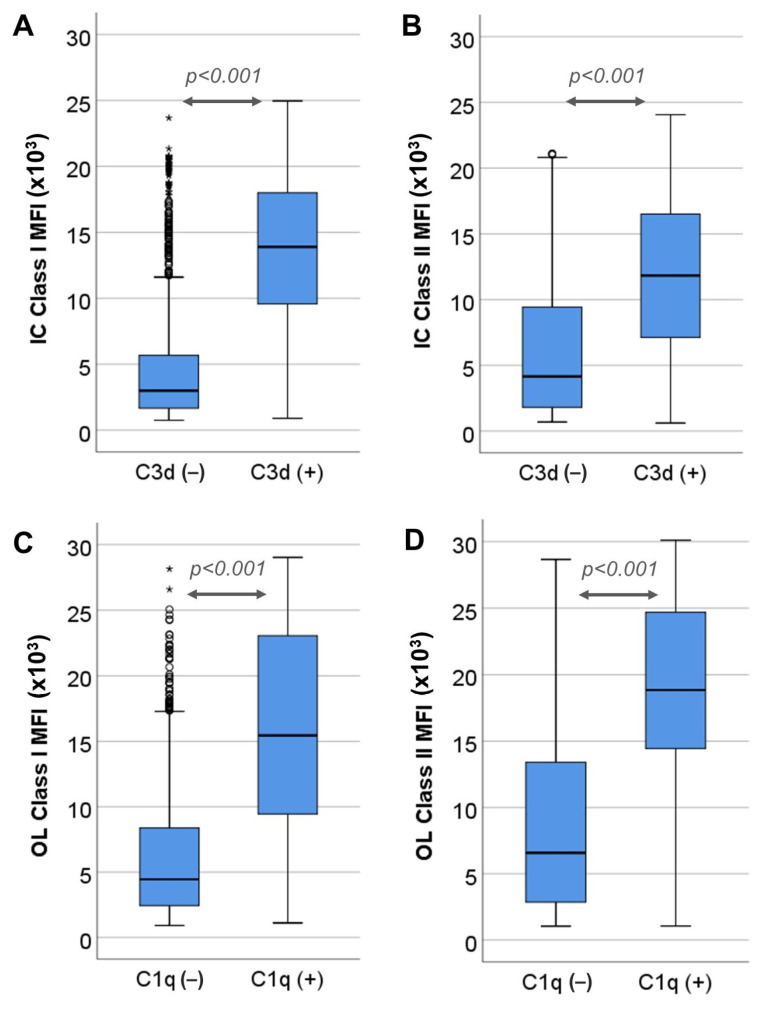
Comparison of MFI values between complement-binding and non-complement-binding specificities. (**A**) MFI values of non-complement binding (C3d−) and complement binding (C3d+) class I positive specificities determined via Immucor (IC). (**B**) MFI values of C3d− and C3d+ class II positive specificities determined via IC. (**C**) MFI values of C1q− and C1q+ class I positive specificities detected via One-Lambda (OL). (**D**) MFI values of C1q− and C1q+ class II positive specificities determined via OL. Circles in the diagram denote outliers laying 1.5–3 times away from the upper limit of the corresponding box. Asterisks denote outliers laying more than 3 times away from the upper limit of the corresponding box.

Complement binding occurred at higher MFI values for both assays. For the IC assay, 428 out of 1608 (26.6%) positive class I specificities bound C3d with a median MFI value of 13,900 (9540–17,999), vs. that of 2991 (1657–5674) for non-complement binding specificities (*p* < 0.001) (Figure 3A), while 409 out of 1136 (36%) positive class II specificities bound C3d with a median MFI of 11,832 (7128–16,531), vs. that of 4152 (1798–9534) for non-complement binding specificities (*p* < 0.001) (Figure 3B).

In the same manner, for the OL assay, 485 out of 1942 (25%) positive class I specificities bound C1q with median MFI value 15,452 (9369–23,095), vs. 4446 (2435–8381) for non-complement binding specificities (*p* < 0.001) (Figure 3C), while 298 out of 1247 (23.9%) positive class II specificities bound C1q with median MFI value 18,852 (14,415–24,707), vs. 6575 (2851–13,412) for non-complement binding specificities (*p* < 0.001) (Figure 3D).

### 3.5. Agreement between the Complement Binding Assays

The number of complement-binding class I specificities did not differ between C3d and C1q assays for N = 30 cases, in which both complement methods detected anti-HLA antibodies (8 (3–14) vs. 8 (4–23) specificities per case for C3d and C1q, respectively; *p* = 0.84). However, the number of complement-binding class II specificities was significantly different between the assays, with C3d and C1q detecting 11 (6–15) and. 8 (3–11) specificities per case, respectively (*p* < 0.001).

Out of 428 class I specificities with positive C3d binding, 305 (71.3%) were also positive with the C1q method, with a median MFI value of 22,921 (6744–26,311) vs. 3756 (1935–9812) for only C3d positive class I specificities (*p* < 0.001). Out of 485 class I specificities with positive C1q binding, 300 (61.9%) were also positive with the C3d method, with a median MFI value of 18,188 (7593–24,849) vs. 6093 (2382–16,476) for only C1q positive class I specificities, (*p* < 0.001).

Out of 409 class II specificities with positive C3d binding, 199 (48.7%) were also positive with the C1q method, with a median MFI value of 25,698 (22,925–27,424) vs. that of 19,406 (4996–24,141) for only C3d positive class I specificities (*p* < 0.001). Out of the 298 class II specificities with positive C1q binding, 215 (72.1%) were also positive with the C3d method, with a median MFI value of 11,406 (5109–21,877) vs. 6720 (3242–19,940) for only C1q positive class I specificities (*p* = 0.02).

### 3.6. Differences between Transplanted and Non-Transplanted Patients

**Figure 4:**
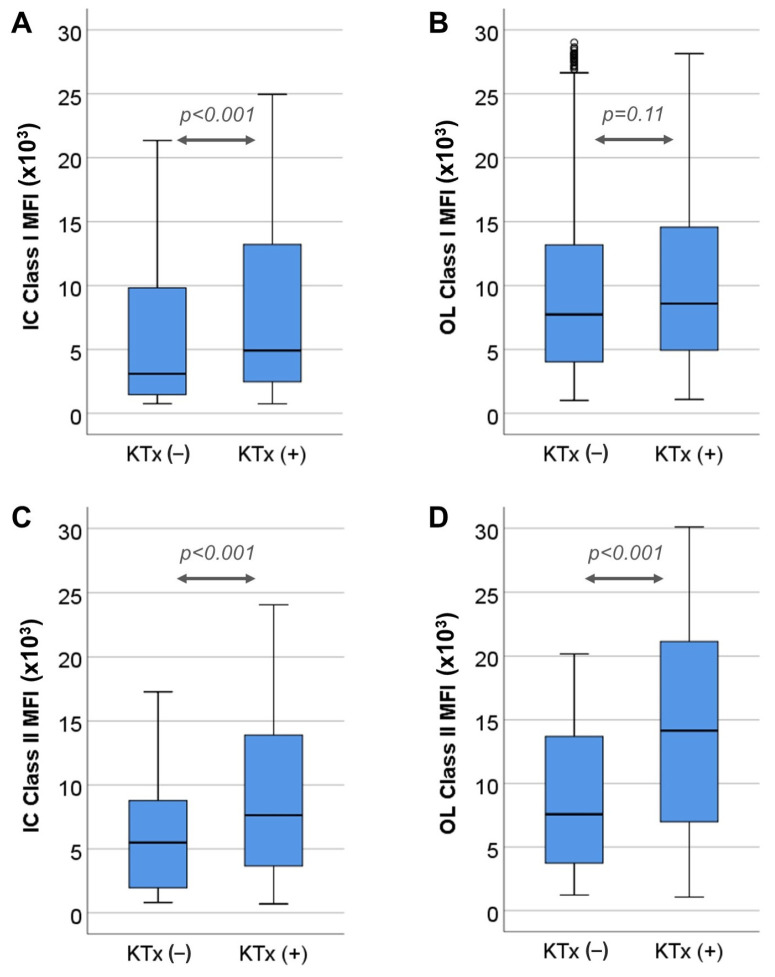
Comparison of MFI values between specificities according to cause of sensitization, (**A**) MFI values of class I specificities determined as positive via Immucor (IC) in non-transplanted (KTx−) and transplanted (KTx+) patients, (**B**) MFI values of class II specificities determined as positive via IC in KTx− and KTx+ patients, (**C**) MFI values of class I specificities determined as positive via One-Lambda (OL) in KTx− and KTx+ patients, (**D**) MFI values of class II specificities determined as positive via OL in KTx− and KTx+ patients. Circles in the diagram denote outliers laying 1.5–3 times away from the upper limit of the corresponding box.

To analyze of the agreement between the methods depending on the cause of sensitization, we compared the MFI values of patients sensitized due to prior kidney transplantation (N = 72) with the MFI values of patients sensitized through other causes (N = 25). This analysis was performed only in the group of patients that had positive specificities with both assays, either class I or class II. In total, 293 positive class I specificities were detected in non-transplanted patients, and 1086 were detected in priorly transplanted patients. The median class I MFI value as measured with IC was lower in non-transplanted patients in comparison to that in prior transplanted patients (3094 (1448–9975) vs. 4910 (2465–13,215), respectively; *p* < 0.001) (Figure 4A), while median class I MFI values did not differ between the two groups when measured with OL (7729 (4010–13,254) vs. 8584 (4934–14,565), respectively; *p* = 0.117) (Figure 4B). Moreover, 78 out of 293 (26.6%) positive class I specificities from non-transplanted patients and 337 out of 1086 (31%) positive class I specificities from transplanted patients bound C3d (chi square = 2.13, *p* = 0.14). Regarding C1q binding, 109 out of 293 (37.2%) positive class I specificities from non-transplanted patients and 322 out of 1086 (29.7%) positive class I specificities from transplanted patients bound C1q (chi square = 6.12, *p* = 0.013). 

Similarly, 136 positive class II specificities were detected in non-transplanted patients and 665 were detected in priorly transplanted patients. The median class II MFI value as measured with both assays was lower in non-transplanted patients compared to that in priorly transplanted ones (IC: 5490 (1960–8796) vs. 7631 (3629–13,920), respectively; *p* <0.001. OL: 7570 (3703–13,726) vs. 14,150 (6988–21,157), respectively; *p* <0.001) (Figure 4C,D). Moreover, 45 out of 136 (33.1%) positive class II specificities from non-transplanted patients and 308 out of 665 (46.3%) positive class II specificities from transplanted patients bound C3d (chi square = 8.02, *p* = 0.005). Regarding C1q binding, 15 out of 136 (11%) positive class II specificities from non-transplanted patients and 225 out of 665 (33.8%) positive class I specificities from transplanted patients bound C1q (chi square = 27.9, *p* < 0.001). Finally, differences in MFI values were analyzed with the two-way ANOVA test, according to complement binding in transplanted and non-transplanted patients, as shown in Supplementary Figure S1. In general, non-transplanted patients had lower MFI values in both assays for class I and II anti-HLA antibodies, irrespective of complement binding, except for C1q binding class I anti-HLA antibodies, which did not differ among transplanted and non-transplanted patients.

### 3.7. PCA Analysis

**Figure 5:**
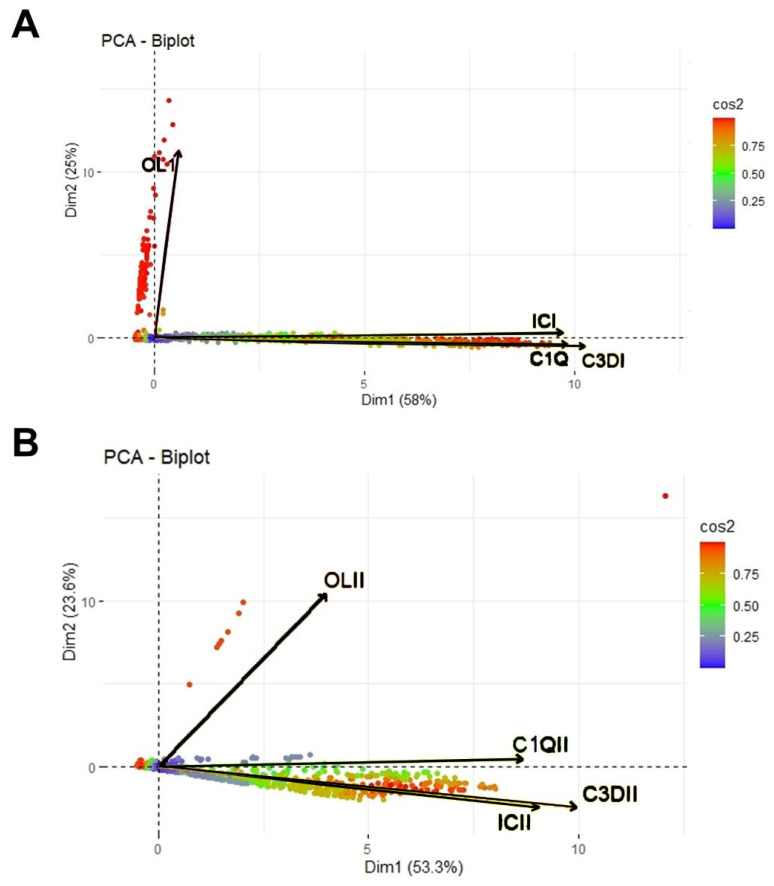
(**A**) PCA biplot of MFI values of class I specificities as determined via Immucor (IC) and One-Lambda (OL), as well as MFI values of complement binding assays, C3d and C1q, on a plane defined by the first two eigenvectors of the covariance matrix. (**B**) PCA biplot of MFI values of class II specificities as determined via IC and OL, as well as MFI values of complement binding assays, C3d and C1q, on a plane defined by the first two eigenvectors of the covariance matrix. Each point represents one single specificity (positive or negative), and arrows depict the corresponding variables. The color of each point is proportional to the cos2 of the explained variance according to the color vector on the right side of the figure. The length of each arrow is proportional to the cos2 of the explained variance.

The first two PCA dimensions captured the high variance of the matrix and showed that the vectors corresponding to screening reactions with the reagents from IC are collinear with the vectors corresponding to reactions observed for complement detection reactions, whichever complement reaction kit is used. The same pattern was observed for both class I and class II reagents, suggesting higher sensitivity for OL reagents, which is not always correlated with complement-binding anti-HLA antibodies (Figure 5).

## 4. Discussion

In this study, we compared the diagnostic performance of IC and OL in the detection of anti-HLA antibodies, and we further characterized the complement-binding capacity of the antibodies detected using each method. According to our results, OL was more sensitive, since it detected a greater number of positive specificities for both anti-HLA class I and II antibodies, when compared to IC. Furthermore, the number of specificities detected via OL only was greater for both anti-HLA class I and class II antibodies, when compared to that detected via IC. Regarding complement binding capacity, both methods were equally effective in detecting complement-binding anti-HLA class I antibodies, whereas the C3d assay was more sensitive in detecting complement-binding anti-HLA class II antibodies. Also, the number of positive anti-HLA class I and II specificities was greater in transplanted compared to non-transplanted patients, and MFI values of anti-HLA class II antibodies were higher in transplanted versus non-transplanted patients when both IC and OL were used. MFI values of anti-HLA class I antibodies were greater in transplanted versus non-transplanted patients, according to IC, but not to OL.

Our study found that OL was more sensitive than IC in terms of both the total number of and unique anti-HLA class I and II antibody specificities. Bertrand et al. also compared the two assays in 100 kidney transplant recipients. The results revealed that OL was more sensitive than IC in detecting AMR (88% vs. 78%), with cutoff values of the MFI for detecting a positive result being 500 for both OL and IC. In order to make the two assays more comparable to each other, the authors plotted receiver operating characteristic (ROC) curves to find the best cutoff value of the MFI. They concluded that an MFI threshold of 2705 for OL and of 473 for IC provided the best cutoff values for making the two assays comparable to each other (with a sensitivity of 80% and 86%, respectively) [15]. Next, Clerkin et al. assessed 125 pre- and post-transplant lung and heart graft recipients. Most HLA class I (94.5%) and HLA class II (89%) antibodies with moderate to high MFI titers (≥4000) were detected via both assays, with a reasonable correlation being detected between MFI values of both assays for both class I and class II antibodies [16]. Since less extensive research exists around the IC assay in detecting anti-HLA antibodies, many investigators use pre-defined cutoff values to make comparisons and determine the positivity threshold. Also, differences in the instructions provided by the manufacturers, such as the lower ratio of patient serum-to-test beads used in IC compared to that used in OL, might account for a dilution-type effect that negates the prozone phenomenon, but leads to reduced sensitivity [17]. In our analysis, we did not use different cutoff values for generating and comparing the sensitivities between the two assays. In the future, however, more research needs to be conducted, since currently there is no consensus regarding the optimal threshold for detecting positive anti-HLA antibody specificities. One way to overcome the lack of consensus regarding the cutoff values for positivity is to further analyze the nature of these antibodies, such as by evaluating their complement binding capacity.

Complement activation is one of the major mechanisms that may result in graft AMR. Detecting complement-activating dnDSAs may be more clinically significant than routine tests, and limiting dnDSAs to only the complement-binding ones could potentially widen the donor pool [12]. According to our results, both complement binding assays were equally effective in detecting anti-HLA class I antibodies, but the C3d assay was more sensitive in detecting complement-binding anti-HLA class II antibodies. Comoli et al. assessed 39 pediatric kidney transplant recipients and found that the presence of C3d-binding dnDSAs could better predict graft loss when compared to that of C1q-fixing dnDSAs [18]. The C3d assay was also used by Sicard et al., who studied 69 kidney transplant recipients with AMR. The results revealed that C3d-binding dnDSAs were associated with a higher risk of graft loss, when compared to C4d graft deposition [19]. C4d was historically known to be the gold-standard test for AMR diagnosis, but reports question whether or not it can satisfactorily predict AMR cases compared to newer methods [20]. Also, since C4d detection relies on tissue specimen inspection, inherent risks associated with the biopsy procedure need to be considered. The improved sensitivity of the C3d assay compared to that of the C1q assay and C4d staining might be explained by tracking the complement cascade pathway. Since C3d is a later product of the complement pathway, it is more indicative of complement activation. The C1q assay, on the other hand, tests the binding of an antibody on an exogenous, purified human C1q molecule, thereby not giving any information as to whether or not downstream complement activation occurs [18,21]. Another topic of controversy that needs to be addressed is the observation of whether or not the complement binding capacity of an antibody is an independent predictor of graft survival or a mere reflection of the amount of the antibody detected (i.e., the MFI value). Several studies have corroborated the above association [2,22]. Complement binding is proportional to antibody titers observed, and in our analysis, the C1q- and C3d-binding anti-HLA class I and II specificities had a higher MFI value compared to that of non-complement-binding specificities. Lan et al. assessed 160 kidney transplant recipients and found a strong correlation between the IgG MFI value and complement-binding dnDSAs, with an MFI value >10,000 yielding a positive C3d result, and the authors concluded that the C3d assay may not have additional discriminatory capacity for detecting AMR when the MFI value exceeds the cutoff value for a positive result. Interestingly enough, however, C3d positivity was also observed in patients with low–moderate MFI values and conferred worse graft survival [23]. Lee et al. also showed that no clear cutoff value could be determined for the MFI and C3d-positive dnDSAs against HLA class II [24]. These results may indicate that the relationship between complement binding capacity and antibody strength might not be absolute, and that the ability of dnDSAs to bind complement elements may be an additional qualitative measure of predicting graft failure. Finally, our results revealed that specificities that bound both C3d and C1q elements had a higher MFI value than that of specificities that bound either complement element alone. Whether or not double positivity for complement elements C3d and C1q results in a worse long-term prognosis and the chance of graft failure is not yet known, and we did not correlate our results clinically, so future studies are required to ascertain whether or not there is a synergistic association between the number of complement fragments binding to an antibody and the risk of AMR.

Apart from inferential statistics, PCA was used to obtain an overview of the trends of both positive and negative specificities. It was of great interest that, overall, the two complement assays appeared to be collinear to IC, delineating consistency among these three methods, while the loading representing OL was perpendicular to that of the others, suggestive of a lower degree of correlation. As shown above, OL detected more specificities than IC did for both class I and II, but especially for class I anti-HLA antibodies. These additional specificities are potentially those that divert OL loading in relationship to the other methods, as they had a lower MFI than the ones detected via both assays, and consequently had a lower chance of complement binding. This difference delineates the significance of complement assays, which are able to identify specificities that are clinically relevant. However, when considering specificities determined as positive with both assays, their MFI values were highly correlated. 

Regarding study limitations, there was no correlation of our findings with the patients’ clinical outcomes, so we could not comment on whether or not detection of complement-binding antibodies is associated with better or worse survival after transplantation. Also, the cutoff values for the detection of anti-HLA antibodies in the two assays were pre-defined by the laboratory that ran the analysis since there is no consensus regarding the optimal threshold for antibody positivity according to MFI values. Finally, we assessed the development of IgG antibodies against HLA class I and II antigens, with no distinction of the specific subtype detected. Specific IgG subclasses have been associated with worse clinical outcomes after transplantation, and this may be related to the MFI value or complement binding capacity of subclass-specific anti-HLA antibodies [25].

## Figures and Tables

**Table 1:** Baseline characteristics of the patients included in the study.

Study patients (N)	97
Age (mean ± SD)	29 ± 13
Sex (male)	50 (52%)
Transplanted, number (%)	72 (74.2%)
Live donor, number (%)	42 (43.2%)
Deceased donor, number (%)	26 (26.8%)
Live and deceased donor, number (%)	4 (4.1%)

Abbreviations list: N: number; SD: standard deviation.

## Data Availability

Data sharing is available and can be provided from the corresponding author upon reasonable request.

## Supplementary Materials

The following supporting information can be downloaded at https://www.mdpi.com/article/10.3390/jcm12247733/s1. Table S1: Common beads between Immucor (IC) and One-Lambda (OL) assays that were included in the statistical analysis; Table S2: Linear correlation between positive specificities detected via Immucor (IC) and One-Lambda (OL) assays; Figure S1. Comparison of MFI values between complement-binding and non-complement binding specificities, according to the cause of sensitization. A. MFI value of non-complement binding (C3d−) and complement binding (C3d+) class I positive specificities detected via Immucor (IC) in non-transplanted (KTx-) and transplanted (KTx+) patients, B. MFI value of C1q− and C1q+ class I positive specificities detected via One-Lambda (OL) in non-transplanted (KTx−) and transplanted (KTx+) patients, C. MFI value of C3d− and C3d+ class II positive specificities detected via IC in non-transplanted (KTx−) and transplanted (KTx+) patients, D. MFI value of C1q− and C1q+ class II positive specificities detected via by OL in non-transplanted (KTx−) and transplanted (KTx+) patients. Circles in the diagram denote outliers laying 1.5-3 times away from the upper limit of the corresponding box. Asterisks denote outliers laying more than 3 times away from the upper limit of the corresponding box
