# Supplementary material for: Complement and Non-Complement Binding Anti-HLA Antibodies Are Differentially Detected with Different Antigen Bead Assays in Renal Transplant Recipients
Source: J Clin Med. 2023 Dec 17;12(24):7733. doi: 10.3390/jcm12247733 (PMC10744102; doi:10.3390/jcm12247733)
Supplement: Supplementary file 1 [file jcm-12-07733-s001.zip › jcm-2716404-supplementary.pdf]

Supplementary Materials

Complement and Non-complement - Binding anti-HLA Antibodies are Differentially Detected with Different Antigen Bead Assays

Contents

<u>Section S1: Supplemental Tables and Figures</u>

Table S1: Common beads between Immucor (IC) and One-Lambda (OL) assays that were included in the statistical analysis

Table S2: Linear correlation between positive specificities detected by Immucor (IC) and One-Lambda (OL) assays

Figure S1. Comparison of MFI values between complement-binding and non-complement binding specificities, according to the cause of sensitization. **A.** MFI value of non-complement binding (C3d-) and complement binding (C3d+) class I positive specificities by Immucor (IC) in non-transplanted (KTx-) and transplanted (KTx+) patients, **B.** MFI value of C1q- and C1q+ class I positive specificities by One-Lambda (OL) in non-transplanted (KTx-) and transplanted (KTx+) patients, **C.** MFI value of C3d- and C3d+ class II positive specificities by IC in non-transplanted (KTx-) and transplanted (KTx+) patients, **D.** MFI value of C1q- and C1q+ class II positive specificities by OL in non-transplanted (KTx-) and transplanted (KTx+) patients.

Section S1: Supplemental Tables and Figures

Table S1: Common beads between Immucor (IC) and One-Lambda (OL) assays that were included in the statistical analysis

HLA class I			HLA class II		
A*01:01	B*07:02	C*01:02	DPA1*01:03	DQA1*01:01	DRB1*01:01
A*02:01	B*08:01	C*02:02	DPA1*02:01	DQA1*01:02	DRB1*01:02
A*02:03	B*13:02	C*03:03	DPA1*02:02	DQA1*01:03	DRB1*01:03
A*03:01	B*14:01	C*03:04	DPA1*03:01	DQA1*02:01	DRB1*03:01
A*11:01	B*14:02	C*04:01	DPA1*04:01	DQA1*03:01	DRB1*03:02
A*11:02	B*15:01	C*05:01	DPB1*01:01	DQA1*03:02	DRB1*04:01
A*23:01	B*15:02	C*06:02	DPB1*02:01	DQA1*04:01	DRB1*04:02
A*24:02	B*15:03	C*07:02	DPB1*03:01	DQA1*05:01	DRB1*04:03
A*24:03	B*15:12	C*08:01	DPB1*04:01	DQA1*06:01	DRB1*04:04
A*25:01	B*15:13	C*14:02	DPB1*04:02	DQB1*02:01	DRB1*04:05
A*26:01	B*15:16	C*15:02	DPB1*05:01	DQB1*02:02	DRB1*07:01
A*29:01	B*18:01	C*16:01	DPB1*06:01	DQB1*03:01	DRB1*08:01
A*29:02	B*27:05	C*17:01	DPB1*09:01	DQB1*03:02	DRB1*09:01
A*30:01	B*27:08		DPB1*11:01	DQB1*03:03	DRB1*10:01
A*31:01	B*35:01		DPB1*13:01	DQB1*04:01	DRB1*11:01
A*32:01	B*37:01		DPB1*14:01	DQB1*04:02	DRB1*11:04
A*33:01	B*38:01		DPB1*15:01	DQB1*05:01	DRB1*12:01
A*33:03	B*39:01		DPB1*17:01	DQB1*05:02	DRB1*12:02
A*34:02	B*40:01		DPB1*18:01	DQB1*06:01	DRB1*13:01
A*36:01	B*40:02		DPB1*19:01	DQB1*06:02	DRB1*13:03
A*43:01	B*41:01		DPB1*28:01	DQB1*06:03	DRB1*14:01
A*66:01	B*42:01			DQB1*06:04	DRB1*15:01
A*66:02	B*44:02				DRB1*15:02
A*68:01	B*44:03				DRB1*15:03
A*68:02	B*45:01				DRB1*16:01
A*69:01	B*46:01				DRB1*16:02
A*74:01	B*47:01				DRB3*01:01
A*80:01	B*48:01				DRB3*02:02
	B*49:01				DRB3*03:01
	B*50:01				DRB4*01:01
	B*51:01				DRB5*01:01
	B*52:01				DRB5*02:02
	B*53:01				
	B*54:01				
	B*55:01				
	B*56:01				
	B*57:01				
	B*58:01				
	B*59:01				

B*67:01
B*73:01
B*78:01
B*81:01

Abbreviations list: HLA: human leukocyte antigen.

Table S2: Linear correlation between positive specificities detected by Immucor (IC) and One-Lambda (OL) assays

Bead	N	Spearman Correlation		Bead	N	Spearman Correlation	
		R	*P*			R	*P*
A*01:01	24	0.48	0.017	DRB1*01:01	8	0.31	0.456
A*02:01	24	0.36	0.079	DRB1*01:02	8	0.33	0.420
A*02:03	24	0.29	0.161	DRB1*01:03	10	0.57	0.082
A*03:01	16	0.58	0.018	DRB1*03:01	18	0.87	<0.001
A*11:01	26	0.59	0.002	DRB1*03:02	19	0.81	<0.001
A*11:02	24	0.62	0.001	DRB1*04:01	14	0.70	0.005
A*23:01	26	0.41	0.039	DRB1*04:02	14	0.58	0.029
A*24:02	29	0.61	<0.001	DRB1*04:03	13	0.88	<0.001
A*24:03	29	0.75	<0.001	DRB1*04:04	8	0.85	0.007
A*25:01	25	0.53	0.006	DRB1*04:05	13	0.62	0.022
A*26:01	20	0.49	0.026	DRB1*07:01	21	0.79	<0.001
A*29:01	6	0.14	0.787	DRB1*08:01	13	0.55	0.049
A*29:02	16	0.16	0.546	DRB1*09:01	20	0.52	0.017
A*30:01	15	0.66	0.007	DRB1*10:01	10	0.41	0.229
A*31:01	12	0.49	0.102	DRB1*11:01	8	0.71	0.047
A*32:01	15	0.55	0.031	DRB1*11:04	10	0.78	0.008
A*33:01	14	0.56	0.036	DRB1*12:01	15	0.43	0.106
A*33:03	15	0.67	0.006	DRB1*12:02	8	0.92	0.001
A*34:02	18	0.60	0.008	DRB1*13:01	15	0.90	<0.001
A*36:01	24	0.82	<0.001	DRB1*13:03	13	0.87	<0.001
A*43:01	19	0.72	<0.001	DRB1*14:01	17	0.85	<0.001
A*66:01	21	0.58	0.005	DRB1*15:01	6	0.60	0.208
A*66:02	19	0.39	0.097	DRB1*15:02	5	-	-
A*68:01	26	-0.08	0.668	DRB1*15:03	5	-	-
A*68:02	26	0.27	0.170	DRB1*16:01	5	-	-
A*69:01	24	0.24	0.252	DRB1*16:02	6	0.37	0.468
A*74:01	11	0.44	0.174	DRB3*01:01	18	0.68	0.002
A*80:01	23	0.79	<0.001	DRB3*02:02	9	0.2	0.606
B*07:02	16	0.72	0.002	DRB3*03:01	15	0.60	0.018
B*08:01	20	0.55	0.012	DRB4*01:01	8	0.81	0.015
B*13:02	23	0.52	0.011	DRB5*01:01	12	0.21	0.499
B*14:01	12	0.82	0.001	DRB5*02:02	8	0.66	0.071
B*14:02	13	0.83	<0.001	DQA1*01:01	11	0.04	0.894
B*15:01	15	0.68	0.005	DQA1*01:02	7	0.25	0.589
B*15:02	18	0.69	0.001	DQA1*01:03	8	-0.12	0.778
B*15:03	15	0.69	0.004	DQA1*02:01	10	-0.38	0.276
B*15:12	26	0.53	0.005	DQA1*03:01	15	0.41	0.126
B*15:13	20	0.78	<0.001	DQA1*03:02	17	0.27	0.277
B*15:16	19	0.54	0.018	DQA1*04:01	20	0.18	0.442
B*18:01	14	0.88	<0.001	DQA1*05:01	22	0.08	0.693
B*27:05	29	0.68	<0.001	DQA1*06:01	9	0.18	0.637

B*27:08	18	0.80	<0.001	DQB1*02:01	20	0.16	0.486
B*35:01	15	0.58	0.022	DQB1*02:02	19	0.42	0.069
B*37:01	17	0.67	0.003	DQB1*03:01	18	0.19	0.448
B*38:01	17	0.61	0.009	DQB1*03:02	24	0.53	0.008
B*39:01	6	0.54	0.266	DQB1*03:03	24	0.33	0.106
B*40:01	20	0.73	<0.001	DQB1*04:01	22	0.75	<0.001
B*40:02	22	0.67	0.001	DQB1*04:02	19	0.27	0.250
B*41:01	15	0.76	0.001	DQB1*05:01	13	0.20	0.494
B*42:01	13	0.31	0.297	DQB1*05:02	5	0.1	0.873
B*44:02	17	0.84	<0.001	DQB1*06:01	9	0.13	0.732
B*44:03	20	0.72	<0.001	DQB1*06:02	12	0.09	0.779
B*45:01	20	0.73	<0.001	DQB1*06:03	9	-0.16	0.668
B*47:01	18	0.75	<0.001	DQB1*06:04	10	-0.13	0.701
B*48:01	8	-0.21	0.610	DPA1*01:03	3	-	-
B*49:01	22	0.57	0.006	DPA1*02:01	1	-	-
B*50:01	18	0.59	0.01	DPA1*02:02	3	-	-
B*51:01	21	0.69	0.001	DPA1*03:01	3	-	-
B*52:01	18	0.67	0.002	DPA1*04:01	2	-	-
B*53:01	21	0.65	0.001	DPB1*01:01	7	0.85	0.014
B*54:01	15	0.84	<0.001	DPB1*02:01	4	-	-
B*55:01	14	0.67	0.001	DPB1*03:01	9	0.73	0.025
B*56:01	16	0.68	0.004	DPB1*04:01	2	-	-
B*57:01	29	0.44	0.017	DPB1*04:02	4	-	-
B*58:01	28	0.74	<0.001	DPB1*05:01	9	0.65	0.058
B*59:01	18	0.65	0.004	DPB1*06:01	4	-	-
B*67:01	16	0.22	0.42	DPB1*09:01	4	-	-
B*73:01	8	0.71	0.047	DPB1*11:01	3	-	-
B*78:01	17	0.74	0.001	DPB1*13:01	7	0.75	0.52
B*81:01	14	0.72	0.004	DPB1*14:01	10	0.74	0.013
C*02:02	7	0.84	0.016	DPB1*15:01	3	-	-
C*03:03	9	0.76	0.017	DPB1*17:01	10	0.55	0.098
C*03:04	7	0.75	0.049	DPB1*18:01	6	0.25	0.623
C*04:01	5	0.66	0.219	DPB1*19:01	8	0.69	0.058
C*05:01	7	0.56	0.146	DPB1*28:01	4	-	-
C*06:02	8	0.88	0.001				
C*08:01	4	0.63	0.368				
C*15:02	7	0.32	0.478				
C*17:01	4	0.80	0.200				

Abbreviations list: HLA: human leukocyte antigen.

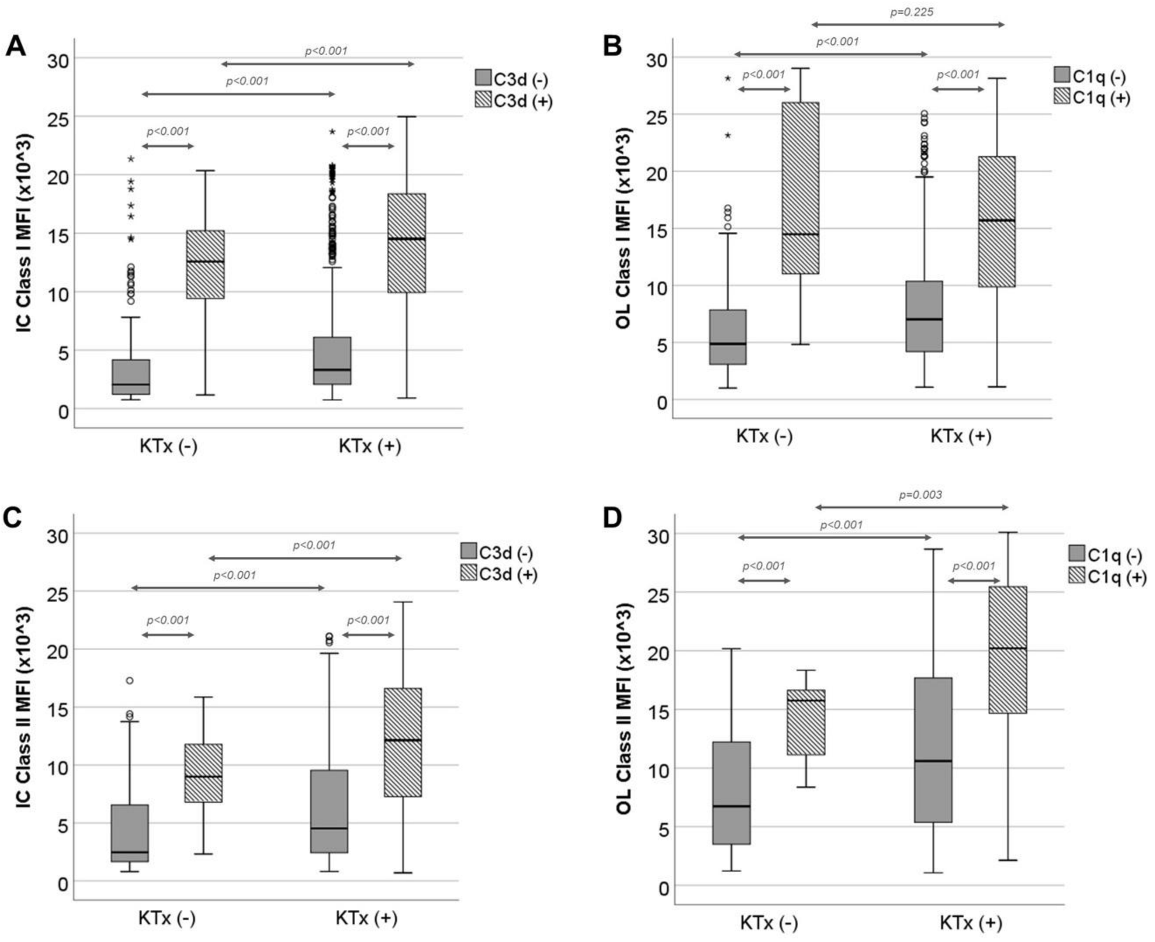

Figure S1. Comparison of MFI values between complement binding and non-complement binding specificities, according to the cause of sensitization. **A.** MFI value of non-complement binding (C3d-) and complement binding (C3d+) class I positive specificities by Immucor (IC) in non-transplanted (KTx-) and transplanted (KTx+) patients, **B.** MFI value of C1q- and C1q+ class I positive specificities by One-Lambda (OL) in non-transplanted (KTx-) and transplanted (KTx+) patients, **C.** MFI value of C3d- and C3d+ class II positive specificities by IC in non-transplanted (KTx-) and transplanted (KTx+) patients, **D.** MFI value of C1q- and C1q+ class II positive specificities by OL in non-transplanted (KTx-) and transplanted (KTx+) patients.
